# No clinically relevant relationship between different quantitative measurement methods of the lateral femoral condyle morphology on lateral radiographs in anterior cruciate ligament‐injured patients

**DOI:** 10.1002/jeo2.70078

**Published:** 2024-11-04

**Authors:** Steffen T. Ubl, Johannes C. Harmes, Evamaria Koch, Arasch Wafaisade, Daniel Guenther, Bertil Bouillon, Thomas R. Pfeiffer

**Affiliations:** ^1^ Department of Orthopaedic Surgery, Trauma Surgery and Sports Medicine, Cologne Merheim Medical Center Witten/Herdecke University Cologne Germany; ^2^ Institute of Interventional and Diagnostic Radiology and Neuroradiology University Hospital Essen Essen Germany

**Keywords:** biomechanic, bone morphometry, femur, geometry, injury, knee, risk factor, rotatory instability

## Abstract

**Purpose:**

To clarify whether different methods of quantifying lateral femoral condyle (LFC) bone morphology as risk factors for anterior cruciate ligament (ACL) injury on lateral radiographs should be considered as individual risk factors and to assess inter‐ and intraobserver reliability.

**Methods:**

We retrospectively reviewed 487 patients undergoing primary ACL reconstruction at our institution. Routine lateral radiographs of the injured knees were utilized to measure the following parameters: LFC ratio (LFCR), height of LFC to anteroposterior diameter ratio (HAPR), femur tibia size ratio (FTSR), tibia to posterior femoral condyle ratio (TPFCR) and Porto ratios (XY/AB; B/AB; B/XY). Malrotated radiographs were excluded. Pearson's correlation coefficients were used to identify relationships. Intraclass correlation coefficients were calculated for inter‐ and intraobserver reliability for two raters.

**Results:**

Fifty‐eight patients were included. Means and standard deviations for LFCR were 63.7% ± 2.8%, HAPR 0.35 ± 0.02, FTSR 1.23 ± 0.07, TPFCR 2.99 ± 0.28, XY/AB 0.41 ± 0.08, B/AB 1.20 ± 0.06 and B/XY 3.05 ± 0.58. Significant correlations were observed between FTSR and XY/AB (*r* = 0.425), B/AB (*r* = 0.582) and TPFCR (*r* = −0.326), between XY/AB and HAPR (*r* = −0.309) and B/XY (*r* = −0.933) and between TPFCR and B/AB (*r* = 0.302). Intraobserver agreement was excellent for LFCR, HAPR, FTSR, TPFCR and B/AB and good for XY/AB and B/XY. Interobserver agreement varied from poor for XY/AB and B/XY, good for HAPR, B/AB, FTSR and TPFCR to excellent for LFCR.

**Conclusion:**

Different methods of quantifying LFC bone morphology should be considered as individual risk factors, characterized by good to excellent intraobserver reliability, but highly variable interobserver reliability.

**Level of Evidence:**

Level III.

**Abbreviations**
ACL: anterior cruciate ligament
ap: anteroposterior
BMI: body mass index
CI: confidence interval
CT: computer tomography
FTSR: femur tibia size ratio
HAPR: ratio of height of lateral femoral condyle to anteroposterior diameter
ICC: intraclass correlation coefficient
LFC: lateral femoral condyle
LFCI: lateral femoral condyle index
LFCR: lateral femoral condyle ratio
MRI: magnetic resonance imaging
TPFCR: tibia to posterior femoral condyle ratio

## INTRODUCTION

Due to the high incidence of anterior cruciate ligament (ACL) injuries [25]⁠ and their vast impact on patients' knee‐related quality of life [20]⁠ and healthcare costs [10]⁠, there is major interest in defining and understanding the risk factors that predispose individuals to primary and secondary ACL tears [1]⁠. Various external and internal risk factors have been investigated with the aim of developing prevention strategies and screening methods for ACL injuries. External factors refer to the patient's environment (e.g., meteorological conditions, surface, footwear). Internal factors describe patient‐specific parameters. Anatomical, biomechanical, hormonal and neuromuscular factors were analysed [11]⁠.

Within the last decade, the bone morphology of the knee joint has gained more attention as a significant risk factor [2]⁠. While the posterior tibial slope is now established as an eminent intrinsic risk factor and widely accepted in daily clinical practice [28]⁠, awareness of the bone morphology of the distal femur is increasing in recent research. In particular, the morphological and morphometric parameters of the lateral femoral condyle (LFC) in the sagittal plane appear to play a substantial role in ACL injuries [8, 22]⁠. An increased posterior femoral condylar depth [12, 14, 22]⁠ or an altered sphericity of the LFC [13, 19]⁠ is associated with a higher risk of primary ACL injury. Regarding sagittal morphology of the LFC, various measurement methods have been described on lateral radiographs [3, 19, 22, 26]⁠, computer tomography scans [9, 14, 16]⁠ and magnetic resonance imaging (MRI) [12, 13, 27]⁠. To date, all these parameters have been considered as separate risk factors and no direct comparison has been made to explore potential relationships. Some of the described ratios use the same measured distances, which could lead to high correlation.

Furthermore, some of the described measurement techniques have shown good to excellent repeatability in the original publications. Unfortunately, analysis of agreement among different raters or repeatability by others than the original describing authors is still lacking for some of these techniques.

Hence, the aim of the study was, first, to compare different morphological risk factors of the LFC for ACL injury on lateral radiographs to clarify whether they should be considered individual risk factors or whether statistical relationships exist. Second, we aimed to validate the described measurement techniques with respect to repeatability. It was hypothesized that, first, no significant correlation between the different morphological risk factors of the LFC would be found and, second, all measurement techniques would show high inter‐ and intraobserver reliability.

This study may lead to a better understanding of the osseous morphology of the LFC as a crucial risk factor for ACL injuries and its impact on biomechanical changes in the knee joint.

## MATERIAL AND METHODS

### Study population

**Figure 1:**
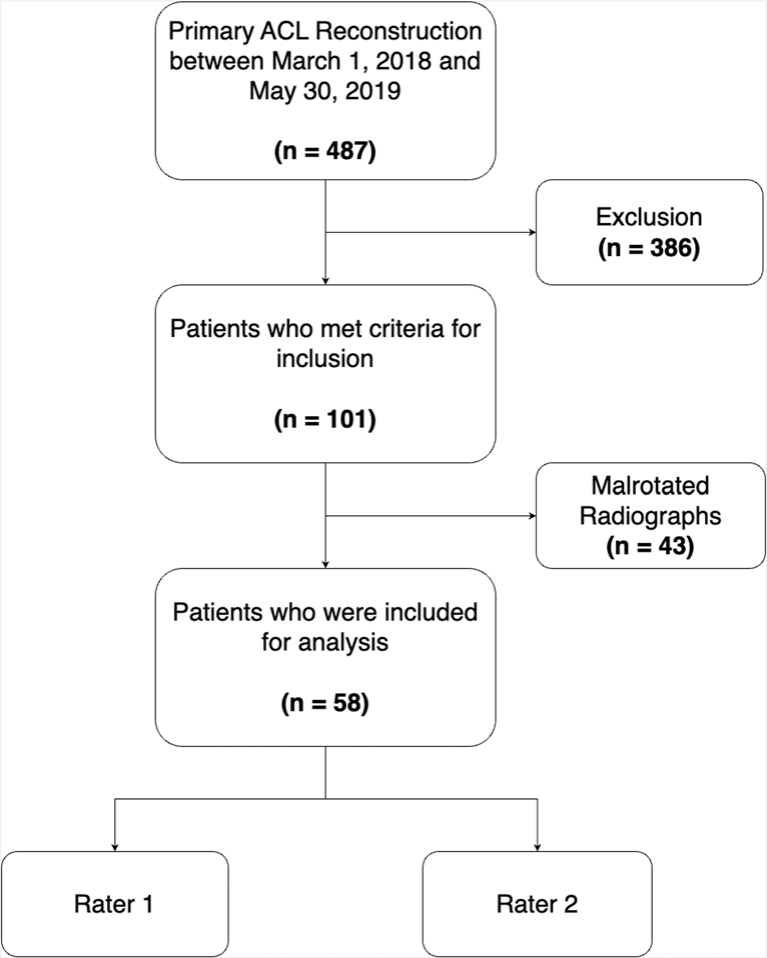
Flowchart of included and excluded patients. ACL, anterior cruciate ligament.

After institutional review board approval, we performed a retrospective analysis of 487 consecutive patients with primary ACL reconstruction at our institution between 1 March 2018 and 30 May 2019. Inclusion criteria were the availability of high‐quality lateral radiographs of the injured knee and no concomitant ligamentous injury. Previous studies suggest that the morphology of the lateral tibiofemoral bone is not a significant risk factor for ACL re‐ruptures in adolescents [7]⁠. In addition, some morphological parameters may change during growth [6]⁠. Therefore, only patients with closed distal femoral and proximal tibial epiphyseal plates were included. As described in a prior study [22],⁠ patients with malrotated lateral radiographs (condylar overlap >6 mm) had to be excluded (Figure 1).

### Measurement methods

**Figure 2:**
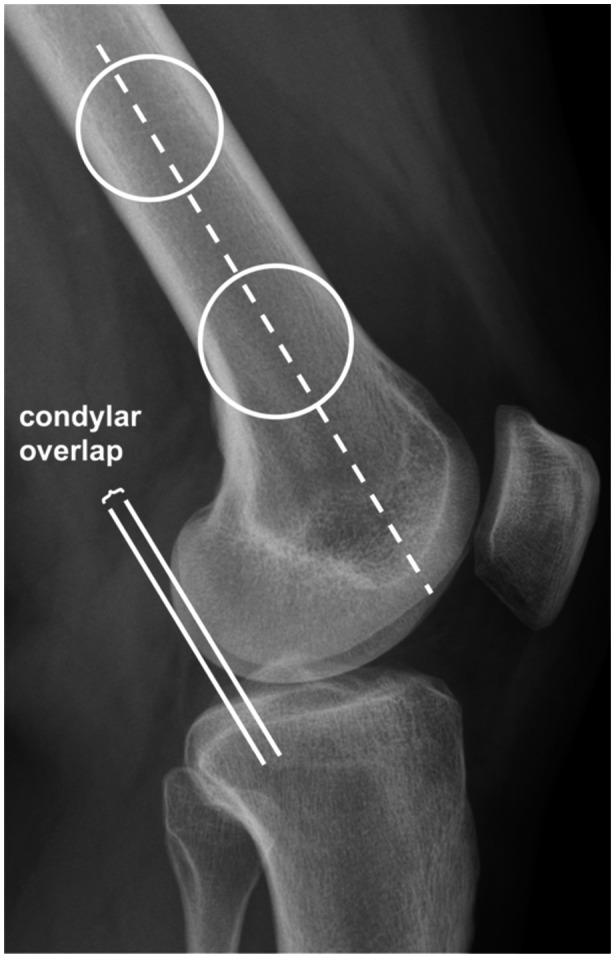
Measurement technique of the condylar overlap. The dotted line represents the longitudinal axis of the femur passing through the centre of two centrally placed circles. Continuous lines are placed parallel to the longitudinal axis of the femur and at the most posterior part of both condyles. The distance between the continuous lines defines the condylar overlap.

Radiographs were imported in DICOM Medical Image Viewer HOROS (Version 4.0.0, Nimble Co LLC d/b/a, Purview). Conventional lateral radiographs were utilized to measure the condylar overlap. First, the longitudinal axis of the distal part of the femur was determined. Two circles were placed 5 cm apart and centrally on the femoral shaft, with the distal circle positioned at the most proximal point of the trochlea. An extended line running through the centre of both circles was considered as the longitudinal axis of the distal femoral shaft. Two parallel lines were duplicated and placed on the most posterior part of the medial and LFC. The distance between these two lines was defined as the condylar overlap (Figure 2).

**Figure 3:**
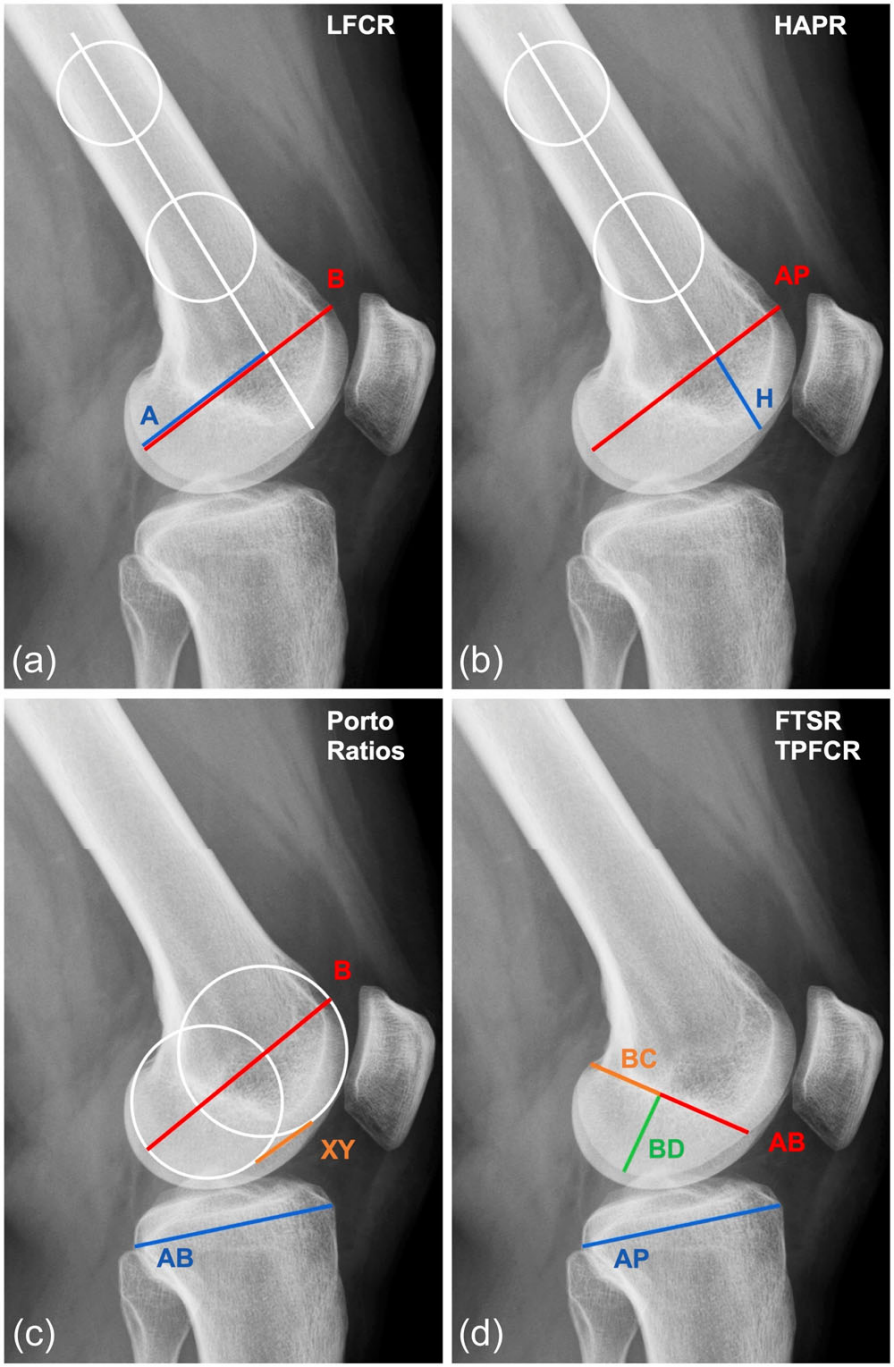
Different quantitative measurement methods of the lateral femoral condyle morphology on lateral radiographs. (a) Lateral femoral condyle ratio (LFCR); [22] (b) Height of lateral femoral condyle to anteroposterior diameter ratio (HAPR); [19] (c) Porto ratios (XY/AB, B/AB, B/XY); [26] (d) Femur tibia size ratio (FTSR) and tibia to posterior femoral condyle ratio (TPFCR) [3].

The following morphometric parameters were measured with the same routine lateral radiographs as described in the original publications (Figure 3): LFC Ratio (LFCR) [22]⁠, Height of LFC to Anteroposterior Diameter Ratio (HAPR) [19]⁠, Porto Ratios (XY/AB; B/AB; B/XY) [26]⁠, Femur Tibia Size Ratio (FTSR) and Tibia to Posterior Femoral Condyle Ratio (TPFCR) [3]⁠.

All measurements were performed by two blinded reviewers. To assess intraobserver reliability, all patients were measured in random order by the main rater (S. T. U.). Six weeks later, the same rater performed all measurements again in random order. To determine interobserver reliability, a second blinded rater (J. C. H.) performed the same measurements on the same patients.

Measured distances are given in millimetres and calculated ratios are shown according to the original publications for better comparability.

### Statistical analysis

SPSS Software Version 29.0.1.0 (IBM Corp.) was used for statistical analysis. Descriptive statistics including means, standard deviations, minimums and maximums were calculated for all continuous variables including age, body mass index (BMI); LFCR; HAPR; XY/AB, B/AB, B/XY; FTSR and TPFCR. Quantitative data were presented as mean ± standard deviation. Pearson's correlation coefficients (*r*) were used to determine whether the morphometric parameters should be considered as individual risk factors or whether statistical relationships existed, with significance set at *p* < 0.05. To reduce the risk of bias and overestimation of small relationships, only correlations with a Pearson correlation coefficient of *r* ≥ 0.6 were considered clinically relevant, as some of the ratios described use the same measured distances. Intraclass correlation coefficients (ICC) and a 95% confidence interval were calculated for inter‐ and intraobserver reliability using an absolute‐agreement, two‐way mixed‐effects model between the two raters. Furthermore, a subgroup analysis of three groups based on condylar overlap was performed. The study population was divided into group 1: 0–1.9 mm, group 2: 2–3.9 mm and group 3: 4–6 mm. This is because initially only LFCR and HAPR were analysed on slightly rotated radiographs with a condylar overlap up to 6 mm. The inter‐ and intraobserver reliability were calculated separately for the three groups. Calculated values were interpreted according to Koo and Li [15]⁠ as poor (ICC < 0.50), moderate (ICC = 0.50–0.75), good (ICC = 0.75–0.90) and excellent (ICC ≥ 0.90). A cutoff value of ICC ≥ 0.7 was defined as acceptable.

A priori sample size calculation was performed using G*Power (Version 3.1.9.7). A high effect size of *f* = 0.50 was expected as some measurement methods use the same measurement distances. The sample size calculation estimated that 46 patients would need to be included to achieve a power of 0.95.

## RESULTS

Fifty‐eight patients (20 females, 28.4 ± 11.6 years old and 38 males, 29.1 ± 12.4 years) were included in the final analysis. The mean BMI was 26.2 ± 5.1. The mean LFC morphology measurements were 63.7 ± 2.8 (LFCR), 0.34 ± 0.02 (HAPR), 0.41 ± 0.08 (XY/AB), 1.20 ± 0.06 (B/AB), 3.05 ± 0.58 (B/XY), 1.23 ± 0.07 (FTSR) and 2.99 ± 0.28 (TPFCR). Measured values of the LFC morphology are shown in Table 1.

**Table 1:** Results of morphological measurements.

	Mean ± SD	min	max
LFCR	63.7 ± 2.8	58.8	68.7
HAPR	0.34 ± 0.02	0.30	0.39
XY/AB	0.41 ± 0.08	0.25	0.58
B/AB	1.20 ± 0.06	1.07	1.34
B/XY	3.05 ± 0.58	2.18	4.91
FTSR	1.23 ± 0.07	1.07	1.44
TPFCR	2.99 ± 0.28	2.51	3.78

Abbreviations: FTSR, femur tibia size ratio; HAPR, ratio of height of lateral femoral condyle to anteroposterior diameter; LFCR, lateral femoral condyle ratio; SD, standard deviation; TPFCR, tibia to posterior femoral condyle ratio.

Significant correlations were observed between FTSR and XY/AB (*r *= 0.425), B/AB (*r* = 0.582) and TPFCR (*r* = −0.326). In addition, there were further correlations between the following parameters: XY/AB and HAPR (*r* = −0.309) and B/XY (*r* = −0.933) and between TPFCR and B/AB (*r* = 0.302). No significant correlations were found between the other measurement parameters. LFCR was the only measurement parameter that showed no correlation with other parameters. The results of Pearson's correlation analysis are shown in Table 2.

**Table 2:** Correlations of morphological parameters.^a^

Parameter	LFCR	HAPR	XY‎/AB	B/AB	B/XY	FTSR	TPFCR
LFCR	1	−0.16	0.02	0.07	−0.01	0.19	0.14
HAPR		1	−0.31*	−0.12	0.23	−0.15	−0.11
XY‎/AB			1	0.14	−0.93**	0.42**	0.18
B/AB				1	0.16	0.58**	−0.30*
B/XY					1	−0.24	−0.24
FTSR						1	−0.33*
TPFCR							1

Abbreviations: FTSR, femur tibia size ratio; HAPR, ratio of height of lateral femoral condyle to anteroposterior diameter; LFCR, lateral femoral condyle ratio; TPFCR, tibia to posterior femoral condyle ratio.

^a^ Values are presented as Pearson's correlation coefficients (*r*).

** *p *≤ 0.01.

* *p* ≤ 0.05.

**Figure 4:**
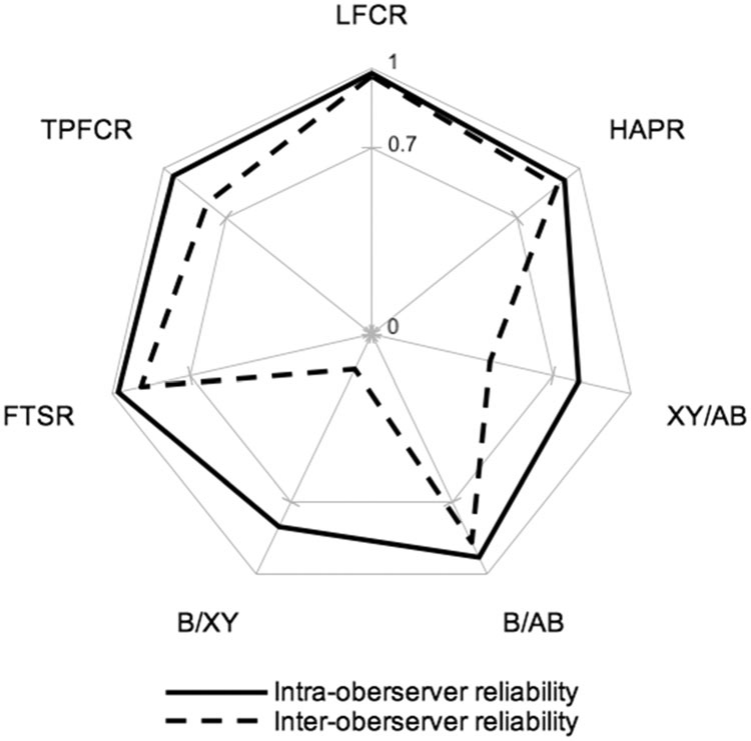
Intraclass correlation coefficients of the intraobserver (continuous line) and intraobserver (dotted line) reliability analysis with respect to the acceptable cutoff value of ≥0.7. FTSR, femur tibia size ratio; HAPR, ratio of height of lateral femoral condyle to anteroposterior diameter; LFCR, lateral femoral condyle ratio; TPFCR, tibia to posterior femoral condyle ratio.

Regarding intraobserver reliability, all measurement methods showed good (ICC = 0.75–0.90) to excellent (ICC > 0.90) repeatability (Table 3 and Figure 4). The interobserver reliability varied from poor for XY/AB and B/XY (ICC < 0.50; *p* > 0.05), good for HAPR, B/AB, FTSR and TPFCR (ICC = 0.75–0.90; *p* < 0.001) to excellent for LFCR (ICC ≥ 0.90; *p* < 0.001) (Table 4 and Figure 4).

**Table 3:** Intraobserver reliability of morphological parameters.^a^

	Total	Subgroup (condylar overlap)		
		0–1.9 mm	2–3.9 mm	4–6 mm
LFCR	0.98** (0.97–0.99)	0.98** (0.95–0.99)	0.98** (0.97–0.99)	0.97** (0.90–0.99)
HAPR	0.93** (0.87–0.96)	0.88** (0.69–0.96)	0.90** (0.77–0.95)	0.99** (0.95–1.00)
XY/AB	0.80** (0.66–0.88)	0.71** (0.24–0.89)	0.88** (0.74–0.95)	0.52 (−0.37 to 0.85)
B/AB	0.93** (0.88–0.96)	0.94** (0.80–0.98)	0.93** (0.86–0.97)	0.88** (0.61–0.96)
B/XY	0.80** (0.67–0.88)	0.56* (−0.09–0.83)	0.90** (0.78–0.96)	0.56 (−0,30–0.86)
FTSR	0.98** (0.96–0.99)	0.98** (0.95–0.99)	0.96** (0.92–0.98)	0.99** (0.95–1.00)
TPFCR	0.95** (0.92–0.97)	0.95** (0.87–0.98)	0.96** (0.91–0.98)	0.92** (0.75–0.98)

Abbreviations: CI, confidence interval; FTSR, femur tibia size ratio; HAPR, ratio of height of lateral femoral condyle to anteroposterior diameter; LFCR, lateral femoral condyle ratio; TPFCR, tibia to posterior femoral condyle ratio.

^a^ Values are presented as intraclass correlation coefficient (95% CI) based on measurements of rater 1 (SU).

** *p* ≤ 0.01.

* *p* ≤ 0.05.

**Table 4:** Interobserver reliability of morphological parameters.^a^

	Total	Subgroup (condylar overlap)		
		0–1.9 mm	2–3.9 mm	4–6 mm
LFCR	0.97** (0.95–0.98)	0.97** (0.91–0.99)	0.97** (0.94–0.99)	0.96** (0.75–0.99)
HAPR	0.89** (0.82–0.94)	0.87** (0.65–0.96)	0.83** (0.65–0.92)	0.97** (0.91–0.99)
XY/AB	0.46** (0.10–0.68)	0.32 (−0.50–0.73)	0.71** (0.38–0.86)	0.04 (−2.79–0.79)
B/AB	0.87** (0.63–0.94)	0.94** (0.80–0.98)	0.86** (0.42–0.95)	0.71* (0.13–0.91)
B/XY	0.15 (−0.35–0.47)	0.05 (−1.12–0.63)	0.42 (−0.18–0.72)	0.11 (−2.67–0.66)
FTSR	0.89** (0.68–0.95)	0.93** (0.45–0.98)	0.81** (0.5– 0.92)	0.89** (0.63–0.97)
TPFCR	0.79** (0.65–0.88)	0.82** (0.50–0.93)	0.79** (0.56–0.90)	0.73* (0.18–0.92)

Abbreviations: CI, confidence interval; FTSR, femur tibia size ratio; HAPR, ratio of height of lateral femoral condyle to anteroposterior diameter; LFCR, lateral femoral condyle ratio; TPFCR, tibia to posterior femoral condyle ratio.

^a^ Values are presented as intraclass correlation coefficient (95% CI).

** *p* ≤ 0.01.

* *p *≤ 0.05.

The mean condylar overlap was 3.1 ± 1.5 mm. Group 1 (condylar overlap: 0–1.9 mm) consisted of *n* = 10 patients, group 2 (condylar overlap: 2–3.9 mm) consisted of *n* = 31 patients and group 3 (condylar overlap: 4–6 mm) consisted of *n* = 17 patients.

In the subgroup analysis, LFCR, HAPR, B/AB, FTSR and TPFCR showed consistently good to excellent intra‐ and interobserver reliability in all three groups (Tables 3 and 4). XY/AB achieved acceptable (≥0.7) intraobserver reliability in groups 1 and 2. From an overlap of >4 mm (group 3), the intraobserver reliability dropped below the acceptable limit of 0.7. Similar results were observed for B/XY, with intraobserver reliability also below the acceptable limit in group 3 (Table 3). In terms of interobserver reliability, XY/AB and B/XY failed to achieve acceptable interobserver agreement in any of the groups (Table 4).

## DISCUSSION

The main finding of this study was that there is no clinically relevant correlation between the different quantitative measurement methods of the LFC bone morphology in ACL‐injured patients. This supports the hypothesis of this study and highlights the importance of considering the different ratios as individual risk factors. Only a few ratios showed low correlations (*r* < 0.6). However, the correlation analysis of FTSR and B/AB approached the cutoff value for clinical relevance. FTSR was calculated by adding the LFC measurements AB, BC and BD (Figure 3) and dividing by the anteroposterior (ap) depth of the tibia. B/AB is the quotient of the ap depth of the LFC and the ap depth of the tibia. Both measures, therefore, describe the relationship between the size of the LFC and the ap distance of the tibial plateau, which explains the correlation in this study. The difference between these ratios is that B/AB measures the ap depth of the LFC, whereas FTSR only describes the extent of the posterior part of the LFC. Only the comparison of XY/AB and B/XY showed a correlation above the cutoff value of *r* = 0.6, which was expected as both ratios describe the ap extent of the flattened surface of the LFC in relation to the ap length of the tibia (AB) or the ap length of the LFC (B).

In the current literature, there are two main hypotheses regarding different bony morphologies of the LFC that are associated with an increased risk of ACL injury. First, a greater depth of the posterior LFC and, second, an asymmetry of the anterior LFC radius compared with the flexion radius. In addition, there are several explanations for their effects on knee joint kinematics.

Regarding an increased depth of the posterior LFC, it has been shown, that a higher LFCR is associated with a greater rotatory knee instability in ACL‐deficient knees [23]⁠. Furthermore, an increased LFCR is associated with a higher incidence of concomitant injury to structures of the anterolateral corner of the knee in patients with noncontact ACL injuries [5, 17]⁠. This may be because an increased posterior depth of the LFC results in a more oval shape. This may result in a greater anisometry and increased length of lateral and anterolateral knee structures, or a smaller contact surface between the femur and tibia and, consequently, resulting in increased rotatory knee laxity near full extension [22, 23]⁠. Furthermore, Hoshino et al. revealed in a three‐dimensional study that a more posterior location of the transcondylar axis, represented as the condyle offset ratio, is associated with a higher anterior tibial translation, which is a major component of the noncontact ACL injury mechanism [14, 24]⁠. In addition, a larger FTSR or smaller TPFCR, indicating a larger LFC compared to the tibial plateau, is a risk factor for rotatory knee instability [3]⁠. However, this study did not find a correlation between LFCR and FTSR or TPFCR. Therefore, the authors believe that an increased LFCR leads to rotatory instability of the knee due to a lack of passive stabilization provided by the lateral and anterolateral structures, whereas altered FTSR and TPFCR lead to rotatory instability due to a smaller contact area between the femur and tibia.

Based on the measurement technique of the LFCR, Li et al. described the HAPR as a risk factor for ACL injury, which represents the spherical shape of the LFC [19]⁠. A smaller HAPR results in a smaller curvature of the LFC and an increased risk for ACL injury. Despite the fact that LFCR and HAPR are both calculated using the ap distance of the LFC, no correlation could be found. This highlights the complexity of LFC bone morphology, particularly in all three dimensions. Based on our study, we propose that a smaller HAPR and a higher LFCR result in different knee kinematics. Specifically, the authors believe that a smaller HAPR results in more anterior tibial translation, whereas a higher LFCR results in more rotational instability, as explained earlier.

Despite this, the sphericity of the LFC may influence rotational stability, resulting in a higher risk for ACL injury [13, 16, 27]⁠. The LFC index (LFCI) is a measurement technique using MRI to assess the sphericity of the LFC [13]⁠. A reduced LFCI indicates that the anterior portion of the LFC is more prominent than the posterior portion, and thus less congruent anterior radius compared to the posterior flexion radius. Hodel et al. claimed that a reduced LFCI leads to more sliding of the flattened anterior portion of the LFC over the lateral tibial plateau, resulting in rotational instability. This is consistent with the opinion of Vasta et al. that a greater curvature of the LFC relative to the tibial ap distance results in rotatory instability. A more prominent anterior portion of the LFC appears to cause a shorter flattened surface of the LFC relative to the tibial ap distance [8].⁠

Biomechanical studies that support these assumptions are still lacking. Future studies should focus on precisely characterising the influences of different bone morphologies of the LFC on knee kinematics, especially regarding the anterior tibial translation and rotatory knee laxity in intact and ACL‐deficient knees.

Regarding our second hypothesis, the analysis of intraobserver reliability showed good to excellent agreement for all measurement techniques. Interobserver reliability was highly variable. While XY/AB and B/XY did not achieve sufficient interobserver repeatability, LFCR was the only technique to reach excellent agreement.

As only LFCR and HAPR were described for malrotated images with up to 6 mm condylar overlap, a subgroup analysis was performed. This showed that the repeatability of the XY/AB, B/AB, B/XY decreased from an overlap of >4 mm. As mentioned by Vasta et al., repeatability tests were not performed in their original publication [26]⁠. Dietvorst et al. investigated the XY/AB, B/AB, B/XY as risk factors for ipsilateral graft rupture and contralateral ACL rupture after ACL reconstruction in children and adolescents using MRI images and found good to excellent repeatability [7]⁠. Thus, our study represents the first repeatability analysis of the XY/AB, B/AB, B/XY as originally described using lateral radiographs [8, 26]⁠.

It appears that the ratios involving the XY distance have an unacceptable interobserver reliability (<0.7). The XY distance is predominantly influenced by the most inferior points of the best‐fitting circles. It is, therefore, questionable whether these circles can be reliably placed on lateral radiographs. Consequently, it can be postulated that the XY/AB, B/AB, B/XY are not applicable to routine radiographs, which usually show some degree of malrotation. This suggests that this complex measurement may be more appropriate for three‐dimensional imaging, where malrotation can be compensated for. Additionally, as others have already pointed out, the best‐fitting circles have great randomness and uncertainty, especially in determining the inferior points of both circles, which could lead to large measurement errors in this method [18, 21]⁠. According to the authors, measurement methods of the bone morphology of the knee should be as simple as possible to ensure clinical applicability and repeatability.

A strength of this study is the use of radiographs from daily clinical practice. On the one hand, this is essential because only measurement techniques that are reproducible on routine radiographs can potentially be implemented in clinical practice. On the other hand, this is a limitation of our study because a large number of patients had to be excluded due to malrotated radiographs. The strict inclusion criteria also limited the number of patients included in the final analysis to *n* = 58. However, enough patients were enroled to meet our a priori power analysis. As mentioned above, our study is the first to validate the XY/AB, B/AB, B/XY using lateral radiographs. Acceptable repeatability could not be demonstrated. This also limits the conclusiveness of our study in terms of correlation analysis of the ratios XY/AB, B/AB and B/XY. Furthermore, the sample size for the subgroup analysis in group 1 was quite small (*n* = 10). However, the minimum sample size required to estimate an ICC value of 0.7 and a power of 0.8 is *n* = 10 [4]⁠ and was, therefore, considered adequate for this subgroup analysis.

## CONCLUSION

No correlation was found between all methods, despite their individual associations with an increased risk of ACL injury or rotatory knee instability. Thus, the complex morphology of the LFC can be quantified using various methods on two‐dimensional, lateral radiographs, with the LFCR showing excellent intra‐ and interobserver reliability, making it suitable for daily clinical use.

## AUTHOR CONTRIBUTIONS

All authors contributed to the study conception and design. Material preparation and data collection were done by Steffen T. Ubl, Evamaria Koch and Johannes C. Harmes. Measurements were performed by Steffen T. Ubl and Johannes C. Harmes. The first draft of the manuscript was written by Steffen T. Ubl, and Thomas R. Pfeiffer made meaningful corrections to the structure of the article and guided the statistical methods and data processing. All authors commented on previous versions of the manuscript. All authors have read and approved the manuscript.

## CONFLICT OF INTEREST STATEMENT

T. R. P. receives research grants from Arthrex outside the submitted work. The remaining authors declare no conflict of interest.

## ETHICS STATEMENT

Approval was obtained from the University of Witten/Herdecke (109/2016).

## Data Availability

The data sets utilized during the current study are available from the first author (ublsteffen@gmail.com) upon reasonable request.
